# Primary somatosensory cortical processing in tactile communication

**DOI:** 10.1098/rstb.2023.0249

**Published:** 2024-07-15

**Authors:** Anne Margarette S. Maallo, Giovanni Novembre, Anikó Kusztor, Sarah McIntyre, Ali Israr, Gregory Gerling, Malin Björnsdotter, Håkan Olausson, Rebecca Boehme

**Affiliations:** ^1^ Center for Social and Affective Neuroscience, Department of Biomedical and Clinical Sciences, Linköping University, 58183 Linköping, Sweden; ^2^ Center for Medical Imaging and Visualization, Linköping University, 58183 Linköping, Sweden; ^3^ Division of Cell and Neurobiology, Department of Biomedical and Clinical Sciences, Linköping University, 58183 Linköping, Sweden; ^4^ School of Psychological Science, Monash University, Melbourne, Victoria 3168, Australia; ^5^ Department of Systems and Information Engineering, University of Virginia, Charlottesville, VA 22904, USA; ^6^ Department of Affective Psychiatry, Sahlgrenska University Hospital, 41345 Gothenburg, Sweden; ^7^ Center for Cognitive and Computational Neuropsychiatry (CCNP), Karolinska Institute, 17177 Solna, Sweden; ^8^ Reality Labs Research, Meta Platforms Inc., Redmond, WA 98052, USA

**Keywords:** touch, fMRI, communication

## Abstract

Touch is an essential form of non-verbal communication. While language and its neural basis are widely studied, tactile communication is less well understood. We used fMRI and multivariate pattern analyses in pairs of emotionally close adults to examine the neural basis of human-to-human tactile communication. In each pair, a participant was designated either as sender or as receiver. The sender was instructed to communicate specific messages by touching only the arm of the receiver, who was inside the scanner. The receiver then identified the message based on the touch expression alone. We designed two multivariate decoder algorithms—one based on the sender's intent (sender-decoder), and another based on the receiver's response (receiver-decoder). We identified several brain areas that significantly predicted behavioural accuracy of the receiver. Regarding our *a priori* region of interest, the receiver's primary somatosensory cortex (S1), both decoders were able to accurately differentiate the messages based on neural activity patterns here. The receiver-decoder, which relied on the receivers' interpretations of the touch expressions, outperformed the sender-decoder, which relied on the sender's intent. Our results identified a network of brain areas involved in human-to-human tactile communication and supported the notion of non-sensory factors being represented in S1.

This article is part of the theme issue ‘Sensing and feeling: an integrative approach to sensory processing and emotional experience’.

## 1. Introduction

Human touch is a powerful primary social communication channel that enables interpersonal exchange of a broad range of emotional content [1,2]. Touch is particularly relevant for expressing feelings linked to intimate social interactions, such as sympathy and love [3]. How and when touch is used depends on relationship closeness and emotional bonds [4].

Surprisingly few studies have examined communication through touch [1,2]. Pioneering work by Hertenstein and colleagues revealed that humans can recognize emotions from touch alone—both emotions typically considered primary (anger, disgust, fear, happiness, sadness) and even those typically labelled ‘secondary’ (gratitude, love, sympathy) [1,5,6]. These findings were replicated and extended to show that both strangers and romantic partners can successfully recognize a wide range of emotions simply with touch expressions directed to the other person's arm [2,7]. Importantly, for communicating intimate emotions such as love and sympathy, touch is preferred to—and more successful than—other non-verbal forms of communication like facial expressions or body posture [6]. Despite the relevance of touch to social interactions, the neural basis of tactile emotional communication is yet to be closely examined [8].

Previous research on emotion recognition suggests the mirror neuron system and emotional simulation or even contagion as underlying mechanisms [9–12]. In short, these theories suggest that others' emotional states can be understood and empathized by simulating their internal bodily state and their actions and expressions through neural mirroring/simulating (and potentially also physiological mirroring). Through predictive processing the observer can then infer the most likely cause, i.e. emotional state of the observed [13]. Several earlier studies have demonstrated that brain areas previously thought to be ‘primary’ processing areas are activated even when simply observing others' actions and sensations, specifically the motor cortex and the primary somatosensory cortex [14]. Further support for the idea that primary somatosensory cortex (S1) is not only involved in basic somatosensory processing comes from a study showing that the evaluation of affective valence of social touch pictures was disrupted by transcranial magnetic resonance stimulation of S1 [15].

Here, we tested the cortical processing, and particularly the involvement of S1, in communication using touch. We hypothesized that different types of touch intuitively used to communicate different messages would give rise to distinct neural responses, specifically in S1, facilitating human-to-human tactile communication. To test our hypothesis, emotionally close pairs performed a naturalistic touch communication task during functional magnetic resonance imaging (fMRI). One participant was assigned the role of sender and the other the role of receiver. The receiver was placed in the fMRI scanner, and the sender was instructed to communicate six different messages by touching the receiver's left arm. Like in previous studies [1,5], the senders were asked to use intuitive touch patterns. The receiver indicated via button press which message they believed was being communicated in a forced-choice paradigm, and they received no feedback. We then used multivoxel pattern classification analysis to assess the degree to which the communicated messages could be decoded from the response with a focus on S1.

## 2. Material and methods

### (a) Participants

We recruited 23 pairs of emotionally close volunteers. One of the participants in each pair underwent magnetic resonance imaging. Our recruitment criteria were that they felt emotionally close and that they were comfortable with touching each other. All pairs reported to be either close friends or romantic partners. All participants reported to be healthy and to speak English fluently (the task was in English). One pair had to be excluded from the final analysis because they did not follow the task instructions. Another pair ended the experiment early because the person in the scanner experienced claustrophobia. After data pre-processing, we excluded three more pairs owing to excessive motion inside the scanner. Ultimately, we analysed imaging and behavioural data from *n* = 17 pairs (participants in the scanner, mean age: 23.6 ± 3.7 years, range: 19 to 32 years, 9 male and 8 female).

### (b) Task

During fMRI, participants performed the touch-communication-paradigm. The sender tried to communicate in every trial one of six possible messages (attention, calming, gratitude, happiness, love, sadness) to the receiver via touch. These were messages with emotional content that people generally want to communicate using touch [2]. The receiver had to guess which message had been communicated. The couples were introduced to the cues and the sentences describing the messages just before they entered the scanner room [2]. Participants did not know any details about the paradigm before they arrived for their appointment, to avoid the possibility of a planned strategy.

The sender stood next to the MRI scanner and received spoken cues via headphones. These were either a narrative describing the message in a context (e.g. ‘Think of all the wonderful qualities that your partner has, and how they enrich your life. Try to express *love* through touch.’) or just the cue-word (i.e. ‘*love*’). During the first trial for each kind of message, the receiver always heard the whole sentences, and in the following trials of similar messages, the receiver heard only the cues. The presentation of the messages was randomized. After each cue, the receiver heard a countdown (i.e. ‘3, 2, 1, go’), after which they were to perform the touch until they heard a stop signal (i.e. ‘3, 2, 1, stop’). This creates a time window of continuous touch of 10 s. The sender was told to only touch the left forearm of the receiver, which was placed on the receiver's belly, and not to perform any shaking movements, to avoid movement artefacts.

After each touch, the receiver saw a grid of the six possible messages on the screen (arranged in a randomized order). They used a button box in their right hand to choose the message they thought their partner tried to communicate. They had a decision window of 7 s. No feedback was provided. The task consisted of two runs with 60 trials per run. Each message was presented 10 times per run in a pseudorandomized order.

### (c) MRI protocol

The MRI sessions consisted of an anatomical T1 scan, two functional runs during the touch-communication-paradigm, and another anatomical T2 scan. All images were acquired using a 3.0 Tesla Siemens scanner with a 64-channel head coil. T1 images were collected with the following settings: repetition time (TR) = 2300 ms, echo time (TE) = 2.36 ms, flip angle = 8°, field of view = 288 × 288 mm^2^, voxel resolution = 0.87 × 0.87 × 0.90 mm^3^. T2-weighted echo-planar images (EPI) were collected with the following settings: 48 multiband slices (3 slices measured simultaneously), TR = 1030 ms, TE = 30 ms, slice thickness = 3 mm, matrix size = 64*64, field of view = 192 × 192 mm², in-plane voxel resolution = 3 mm², flip angle = 63°.

### (d) Univariate analysis

Functional MRI data were analysed using statistical parametric mapping (SPM12, Wellcome Department of Imaging Neuroscience, London, UK; http://www.fil.ion.ucl.ac.uk/spm) in Matlab R2016a (MathWorks, Natick, MA, USA). The following steps were performed: motion correction, co-registration of the mean EPI and the anatomical image, spatial normalization to the MNI T1 template and segmentation of the T1 image using the unified segmentation approach. Normalization parameters were applied to all EPIs. Finally, all images were spatially smoothed with an isotropic Gaussian kernel of 6 mm full width at half maximum. For statistical analysis of the blood-oxygen-level-dependent (BOLD) response, the general linear model approach was used as implemented in SPM12. Regressors-of-interest were defined separately for each of the messages as perceived by the receiver, convolved with the haemodynamic response function. To account for movement-associated variance, realignment parameters were included as regressors-of-no-interest. The univariate analysis was not our main focus and was only performed to check for main effects.

### (e) Image processing for multivariate analysis

The anatomical image was parcellated according to the Human Connectome Project multimodal atlas [16], and we used combinations of parcels for different brain regions (electronic supplementary material, table S1 and figure S1). Afterwards, the regions of interest (ROIs) were registered to the EPI images and the BOLD time series were obtained from each voxel in a brain region. Using AFNI's afni_proc.py wrapper, the BOLD signal's time series were pre-processed with the standard steps including de-spiking, slice time-correction, volume registration and scaling to a mean of 100. Finally, the functional images were co-registered to the anatomical image (EPI to anatomy). Decoding was carried out on the mean BOLD time series from the primary sensory area described above. Shared data can be found under: https://zenodo.org/uploads/10007018.

## 3. Data analysis

### (a) Decoders

Two different decoders were used: a sender-decoder and a receiver-decoder. Trials for each stimulus were labelled based on either the sender's intent (i.e. the prompt to the sender) or the receiver's interpretation (i.e. the response of the receiver). For example, if a sender was instructed to send a message of ‘love’ and the receiver interpreted it as a message of ‘happiness’, that sender-decoder stimulus was labelled as ‘love’, but the receiver-decoder was labelled as ‘happiness’. Thus, the sender-decoder was considered correct for such a trial if it decoded the response as ‘love’ (regardless of the receiver's interpretation), whereas the receiver-decoder was accurate in the same trial if it labelled the response as ‘happiness’ (regardless of the sender's intent).

For each expression/trial, 10 s observation windows of BOLD signal were collected and labelled for each decoder. The observation window corresponded to the last 5 s while the sender was touching the arm of the receiver and another 5 s afterwards. This summation of the 10 s BOLD signal should capture the peak neural response to the touch, assuming a canonical haemodynamic response function. Each trial was represented by a label (based on either the sender's intent or the receiver's response) and a single value, which was the sum across space and time of the BOLD signal during the 10 s window.

Using Matlab's (mathworks.com) *crossval* function, we cross-validated a multiclass error-correcting output codes (ECOC) model obtained from the *fitceoc* function, which uses support vector machine binary learners. Briefly, an ECOC model reduces the multiclass classification problem into a multi-set of binary classification problem [17]. Lastly, we used *kfoldPredict* to obtain the predicted output from the trained model and compared the prediction with the receiver's behavioural response.

### (b) Modelling behaviour with neural data

A linear model with mean hit rate (behaviour accuracy averaged over all expressions) as the dependent variable and normalized sender- and receiver-decoder performance as predictors was used to test for neural involvement in the performance in the touch communication task. Normalized performance was computed as follows: the average of the difference of each expression's confusion matrix from a perfect matrix was computed for each brain region. For instance, the sender-decoder's confusion matrix for ‘attention’ was subtracted from an ideal matrix with 100% along the diagonal and 0 on all the off-diagonal elements, generating a 6 × 6 distance matrix for that expression. These matrices were averaged over all expressions and normalized. A perfectly accurate decoder would have a value of 1 while a completely inaccurate decoder would have a value of 0. The linear model was of the form: *behaviour* ≈ *sender-decoder accuracy + receiver-decoder accuracy + err* and was compared with a null model (i.e. behaviour had no dependence on decoder accuracy). Results were considered significant if they survived multiple comparison correction (false discovery rate, FDR, at 0.05).

### (c) Statistical significance

We used the statistical significance cutoff based on small datasets as outlined in [18]. For an infinite multiclass classification problem, the theoretical chance level is simply 1/*N*, where *N* is the number of classes. However, with limited trials and number of classes *N* = 6 as is the case here, we used the formula St(*α*) = binoinv(1 – *α*, *T*, 1/*N*) × 100/*T*, where 'binoinv' is the binomial inverse cumulative distribution function and *T* = total number of trials (here, 2 runs × 60 trials = 120). From these values, we obtained a chance level of 22.5% at *α* = 0.05 (as opposed to the simple 1/6 ≈ 16.7% in an infinitely sampled classification problem).

## 4. Results behaviour

**Figure 1.**
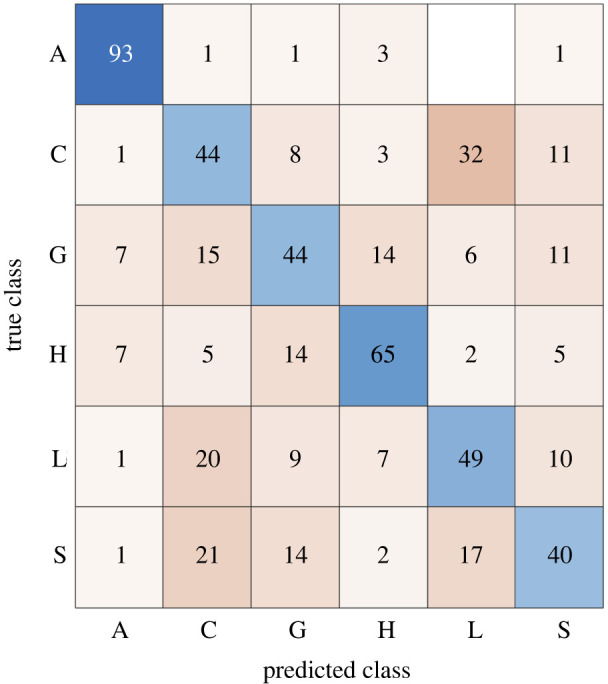
Mean group accuracy in a 6-class touch identification task. The true class is the message that the sender was attempting to convey. The predicted class is the receiver's response. Receivers can accurately identify (values along the diagonal, above chance ≥22.5%) touch messages. Values displayed are rounded; rows do not sum up to 100. A: attention, C: calming, G: gratitude, H: happiness, L: love, S: sadness. Blue/orange: accurate/inaccurate; intensity is proportional to magnitude.

Senders were instructed to communicate the messages with intuitive forms of touch. To this end, the expressions were not controlled across participant pairs. It was at the discretion of the senders alone as to how to convey the message they heard through the prompts. From this, we found above-chance mean group accuracy (≥22.5% with *n* = 120, *α* = 0.05) of behavioural responses, i.e. correct identification, for all six messages communicated through expressions composed of intuitive tactile gestures (figure 1). ‘Attention’ had the highest overall hit rate at 93.9%. These data were previously reported in McIntyre *et al*. [2] as experiment 2 of four, with different and more detailed behavioural analyses, which can be found in McIntyre *et al*. [2, Fig. 1e,f and Fig. 5b] and electronic supplementary material, figure S1*c*.

### (a) Univariate analysis

**Figure 2.**
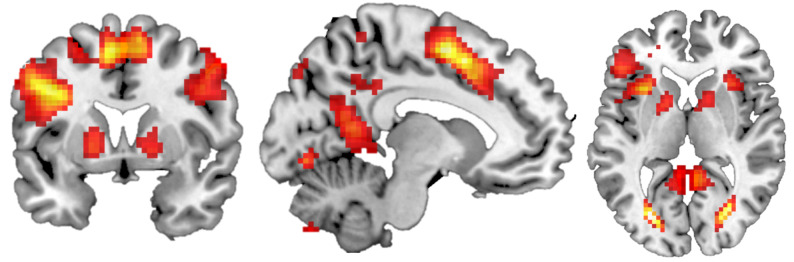
Main effect of touch. Univariate analysis using *F*-test, family-wise-error corrected at the whole brain level, *p* < 0.05, cluster size greater than 20, depicted at [−10 5 4], colour gradient indicates *F*-values (0–100).

We found a main effect of touch in a network of areas including pre- and postcentral gyrus, insula, striatum, supplementary motor area, angular gyrus, occipital and parietal areas, hippocampus and cerebellum (figure 2; electronic supplementary material, table S2). There was no main effect of condition (i.e. perceived cue).

### (b) Multivariate analysis

**Figure 3.**
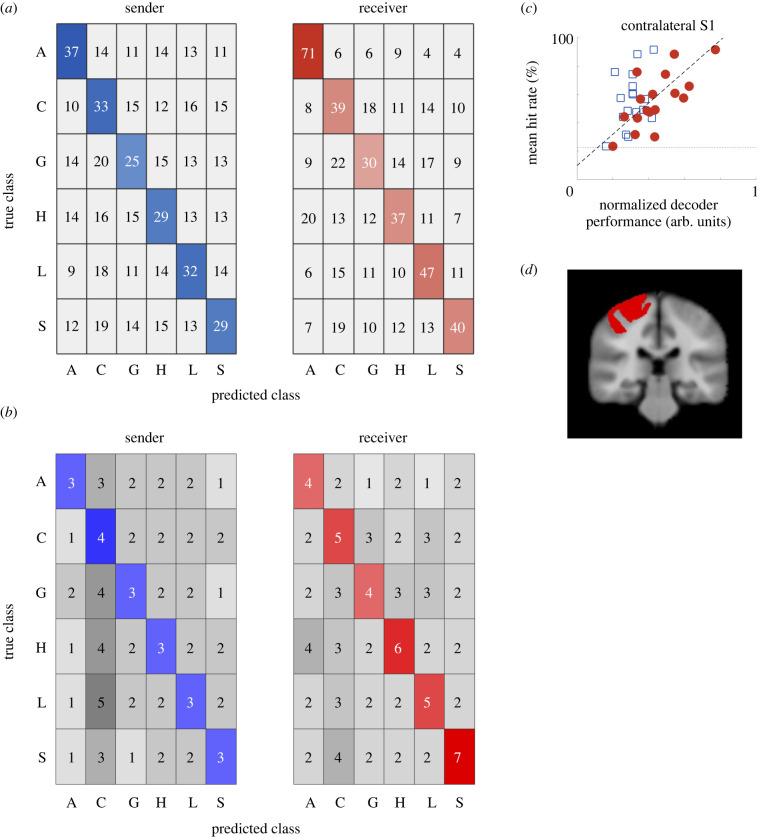
Decoder performance for the region of interest of receivers' contralateral S1. (*a*) From both decoders, accuracy of each expression was higher than chance (≥22.5%), but higher in the receiver- decoder than the sender-decoder. The true class is the message that the sender was attempting to convey (sender-decoder) or the message the receiver recognized (receiver-decoder). The predicted class is the decoders’ prediction. The coloured diagonal therefore displays the decoder's accuracy (%) of identifying the correct touch expression. (*b*) Standard error of the corresponding cells in (*a*) indicates comparable variation for the different touch expression. (*c*) Accurate communication correlated with the receiver's neural decoder. *y*-axis: behavioural performance on the communication task, averaged over all expressions for each dyad. *x*-axis: normalized distance of respective decoder's confusion matrix from an ideal confusion matrix, 1 being closest to ideal and hence indicative of better overall performance. Dotted line: chance accuracy, dashed line: linear fit. Blue squares: sender-decoder data, red circles: receiver-decoder data. (*d*) Para-sagittal slice showing the analysed cortical region corresponding to the right (contralateral) S1.

For both decoders, we found significantly accurate decoding in the receiver's contralateral S1 for each expression (≥22.5% with *n* = 120, *α* = 0.05, according to the method outlined in §3c, ‘Statistical significance’). These accuracies are shown in figure 3 *a* as the values along the diagonals in the confusion matrix. Notably, for each expression, the corresponding accuracy was always higher in the receiver-decoder than in the sender-decoder.

To test the relationship between decoder performance and behaviour in our main ROI, S1, we performed a linear regression of mean hit rate with normalized sender- and receiver-decoder performance as predictors. We found that only the receiver-decoder performance correlated with the hit rate (|*t*| = 3.17, *p* < 0.01, d.f = 14, Cohen's *d* > 1.6; figure 3 *b*, red circles), whereas the sender-decoder did not (|*t*| = 0.33, *p* > 0.7, d.f = 14; figure 3 *b*, blue squares).

Then, we ran an exploratory analysis including all brain regions in order to understand whether this finding was specific for S1 or could be observed also in other brain regions. We therefore compared the regression model with a null model. We found 10 regions with significant difference between this model (table 1) and the null model, including S1 (for full list of all brain regions, see electronic supplementary material, table S3). These effects were driven by the receiver-decoder, i.e. the sender-decoder did not contribute significantly to the model, except for the ipsilateral dorsal visual cortex, where both sender- and receiver-decoder contributed (see electronic supplementary material, figure S2 for correlation depictions).

**Table 1.** Regions that showed statistical significance of model over null. Behaviour modelled as a function of sender- and receiver-decoder performance. *t*-values indicate the contribution of the receiver- and the sender-decoder to the linear regression model. contra: contralateral, ipsi: ipsilateral.

region	*t*-value receiver	*t*-value sender	*F*-value	*p*-value
superior parietal cortex contra	4.08	−0.57	11.8	0.000989
premotor cortex ipsi	4.37	−1.27	10.4	0.00171
inferior frontal cortex ipsi	4.15	−1.27	9.12	0.00291
S1 ipsi	3.87	−1.39	8.76	0.00341
dorsal visual cortex ipsi	2.19	2.4	7.96	0.00491
S1 contra	3.17	0.33	7.17	0.00716
S2 ipsi	2.85	0.77	7.12	0.00738
posterior opercular cortex contra	2.33	0.43	6.98	0.00788
ventral visual cortex ipsi	3.64	−0.22	6.72	0.00899
posterior opercular cortex ipsi	3.13	−1.35	6.62	0.00946

## 5. Discussion

We demonstrated that emotionally close adults could effectively communicate emotional cues using only touch expressions to the forearm and that their success in doing so related to neural processing in several brain areas, including parietal, visual, opercular, premotor and somatosensory areas. Consistent with previous work [1,2,5,6,19], receivers identified the emotions at well above chance level. We further found that the neural response patterns in several areas were distinct enough to elicit above-chance classification accuracy. Overall, the receiver-decoder performed better than the sender-decoder, i.e. the decoder that was based on the receivers' interpretation, not on the senders’ intent.

We previously found evidence that senders, when cued to signal a particular emotion by touch, seem to use generally similar kinds of hand manoeuvres on any given trial [2]. From a purely sensory perspective, the neural responses should be reliably similar when the same expressions are performed repeatedly. By contrast, the neural responses should be distinct for different expressions conveying different messages. Indeed, the sender-decoder achieved an above-chance accuracy in the voxels of S1, suggesting reliably similar expressions eliciting reliably similar neural responses while different expressions elicit different responses, since different types of touch produce distinct patterns of activation starting in peripheral mechanosensitive neurons [20,21]. Notably, the decoders' results were obtained from a large swathe of the cortex that is classified as the primary sensory cortex, and not necessarily constrained to a functionally defined region corresponding to the arm that was being touched. This suggests that, while the cortex is somatotopically organized, there is a broader role of tactile decoding within the entirety of S1.

Interestingly, we found that several brain regions typically not associated with touch processing also were significantly related to performance: visual and premotor areas. The involvement of visual areas can easily be explained: the receivers were to choose the correct answer on the screen afterwards. They might have focused on the word describing their choice—it is possibly that we are picking up this behaviour. Differential activation for the different types of messages in premotor cortex are more surprising. This cannot be explained by the motor activity of choosing the answer, since the position of the different words was varied on the screen (i.e. the motor pattern necessary to choose the answer was randomized). A potential explanation for the involvement of the premotor cortex could be the mechanism of mirroring or simulating described in the introduction: the receiver might have simulated the movement necessary to produce the touch expression, which could relate to or underlie the interpretation.

The other regions that significantly predicted performance have been suggested to be involved in higher level processing of tactile stimuli (S2, operculum, parietal cortex) [22–24]. Parietal cortex has been found to be involved in higher order multisensory processing involving touch-stimuli like touch observation [25] and remapping into external space [26], but also other higher order processing, including language-related processes [16]. The inferior frontal cortex has previously been shown to be involved in touch processing [27] and in the emotional evaluation of observed touch [28]. However, inferior prefrontal cortex has shown involvement in many tasks and its activation might not be specific to the touch here, as it has for example been found to relate to semantic decision making [29] and language processing in general [16].

Within the predictive coding framework, emotions have been suggested as a top-down active inference of causes of interoceptive signals, i.e. physiological changes that may, but do not have to be driven by external events [30]. In our case, the interpersonal touch might be understood as such an external event, which alters the physiological state of the receiver and through this alteration allows an embodied interpretation or understanding of the communicated message. This view is supported by studies showing effects of touch on several physiological parameters, for example breathing frequency, heart rate and its variability, and stress hormone levels (for review see [31]). Furthermore, specifically, light stroking, which is typical for signalling calming or loving emotions [2], activates the insular cortex [32,33] associated with interoception [34]. However, when we modelled behaviour as a function of the sender- and decoder-performance for different regions in the brain, insular cortex was not among the informative regions. Instead, we found other regions in the occipital and parietal lobe, and in the prefrontal cortex, including premotor area, to predict task performance. The lack of insula involvement might be related to the fact that it does not play a crucial role in discrimination of tactile stimuli [35]. Insula is considered to especially process hedonic aspects of touch mediated by C-tactile fibres, which we have shown do not discriminate between different touch expressions [21].

We tried to use emotional messages in our task in order to understand neural communication through touch. We were especially interested in S1 and were able to decode the content of the communicated messages based only on this primary cortex's activation patterns, suggesting that not only the interoceptive changes associated with emotion communication played a role, but that primary sensory processing areas were involved as well. It is important to note that we do not know which strategy our receiver-participants used, i.e. whether they identified the communicated message through sensing an emotional change or through cognition, i.e. reflection, actively classifying and learning the different touch sensation (though no feedback was provided). It has been shown before that S1 is needed for recognizing emotions even if perceived through other senses, e.g. vision [11], and is activated not only when performing an action but also when observing it [14]. A potential underlying mechanism here might be neural mimicry, i.e. the understanding of another person's emotional state through simulation [12]—and potentially experiencing these same emotions through emotional modelling or emotional contagion. While our results might be understood along these lines, it is not possible to differentiate whether the neural decoding accuracy was based on the underlying touch pattern, i.e. differential receptor activations, or indeed on different emotional consequences and potentially physiological changes in the receiver.

The here-differentiated signals might form the basis on which higher order processing areas like the prefrontal cortex or the posterior superior temporal sulcus categorize and identify the emotion [8]. These higher order areas also integrate tactile signals with other sensory signals, since touch occurs in most situations not in isolation, but coupled with multisensory signals. However, one can imagine a situation where early humans had to rely on the sense of touch only (e.g. hiding in a dark cave from a predator). Our findings add to existing literature that shows that interpersonal tactile signals are enough to understand communicative acts: we demonstrate that tactile communication indeed involves S1, although not exclusively.

Several limitations need to be addressed. Our experimental design did not control for the tactile stimulation provided to the receivers. Hence, the resulting brain response patterns may not reflect the same communication patterns, especially between participant pairs. Nevertheless, this may also be considered a strength: since the senders were not restricted to signalling emotions according to a scripted touch expression, we captured the natural variation in human interpersonal touch and our results therefore hold ecological validity. This approach has been successfully used previously [1,5], and our findings therefore complement previous literature. However, since we did not record the touching patterns, we cannot relate the findings to specific sender behaviour, and further studies should investigate the link between the distinctness of the neural patterns and the tactile stimuli (e.g. does the brain respond differently to stroking and tapping emotions?). Similarly, since we did not control for variability in the signalling of specific messages, this limitation restricts any meaningful analysis of the neural correlates of any individual emotion. Our study did not attempt to define the arm representation in S1, and therefore analysis encompassed the whole S1. Lastly, while the effect size in the correlational part of the study is large enough (Cohen's *d* > 1.6), it should still be noted that one limitation of our study is that the sample size was relatively small.

## 6. Conclusion

We were able to successfully identify both the intent and to an even higher degree the interpretation of touch messages based on neural activity in primary somatosensory cortex only. The somatosensory cortex is known to play a key role in social processing and empathy well beyond simple tactile representations [36], including human-to-human shared perception through touch observation [37], as well as identification of affect from felt [38] and observed touch [39]. Specifically, vocal and facial expressions of emotion induce somatosensory cortex responses that discriminate among emotion categories [40]. It may therefore be possible that the observed patterns in S1 go beyond a simple reflection of the peripheral input; however, future research with well controlled tactile stimuli is required to elucidate this question. In sum, these observations highlight the importance of several brain areas, including S1, parietal, opercular, prefrontal and premotor areas, for interpersonal touch communication.

## Data Availability

Data and code can be found at https://zenodo.org/records/10007018 [41]. Supplementary material is available online [42].
